# Modified Lyon brace: with antero-lateral pressure to allow Kyphosis

**DOI:** 10.1186/1748-7161-8-S1-P15

**Published:** 2013-06-03

**Authors:** G Notin, L Journoud, J Deceuninck, C Lecante, F Barral, JC Bernard

**Affiliations:** 1 Lecante company ,Lyon, France; 2 Croix Rouge française CMCR Les Massues, Lyon, France

## Background

In the 50’s, Pierre Stagnara introduced the « lyon treatment ». It included an Abbott plaster cast, followed by a Lyon brace.

## Aim

Can 3D analysis help us today?

## Methods

Lyon braces are designed as Abbott plaster casts. Using a study on plaster cast, and 3D analysis, (called « is Abbot cast still relevant today? » by Dr. Jean Claude Bernard, from the Massues center in Lyon, presented at the SOSORT 2011), we decided to modify a Lyon brace. If a plaster cast is modified, in order to improve sagittal plane by inverting band, and so having antero lateral push in the thoracic part, instead of a classical postero lateral push, the design of the Lyon brace used for the same patient will have an antero lateral pad too.

## Results

The improvement of sagittal plane shown is maintained with the modified Lyon brace

## Conclusion

Introducing 3D analysis, in the design of braces, seems as relevant to maintain sagittal plane as shown last year for plaster cast.
